# The determinants of staff retention after Emergency Obstetrics and Newborn Care training in Kenya: a cross-sectional study

**DOI:** 10.1186/s12913-022-08253-2

**Published:** 2022-07-06

**Authors:** Duncan N. Shikuku, Irene Nyaoke, Onesmus Maina, Martin Eyinda, Sylvia Gichuru, Lucy Nyaga, Fatuma Iman, Edna Tallam, Ibrahim Wako, Issak Bashir, Helen Allott, Charles Ameh

**Affiliations:** 1 Liverpool School of Tropical Medicine, P.O. Box, Nairobi, 24672-00100 Kenya; 2 Department of Health, Garissa, Kenya; 3 Nursing Council of Kenya, Nairobi, Kenya; 4 Clinical Officers Council of Kenya, Nairobi, Kenya; 5 Department of Family Health, Ministry of Health, Nairobi, Kenya; 6 Liverpool School of Tropical Medicine, Liverpool, UK; 7 Department of Obstetrics and Gynaecology, University of Nairobi, Nairobi, Kenya

**Keywords:** Emergency obstetrics and newborn care, Skilled health personnel, Staff retention, Maternal and newborn health, Maternity, Kenya

## Abstract

**Introduction:**

Kenya’s maternal mortality ratio is relatively high at 342/100,000 live births. Confidential enquiry into maternal deaths showed that 90% of the maternal deaths received substandard care with health workforce related factors identified in 75% of 2015/2016 maternal deaths. Competent Skilled Health Personnel (SHP) providing emergency obstetric and newborn care (EmONC) in an enabling environment reduces the risk of adverse maternal and newborn outcomes. The study objective was to identify factors that determine the retention of SHP 1 – 5 years after EmONC training in Kenya.

**Methods:**

A cross-sectional review of EmONC SHP in five counties (Kilifi, Taita Taveta, Garissa, Vihiga and Uasin Gishu) was conducted between January–February 2020. Data was extracted from a training database. Verification of current health facilities where trained SHP were deployed and reasons for non-retention were collected. Descriptive data analysis, transfer rate by county and logistic regression for SHP retention determinants was performed.

**Results:**

A total of 927 SHP were trained from 2014–2019. Most SHP trained were nurse/midwives (677, 73%) followed by clinical officers (151, 16%) and doctors (99, 11%). Half (500, 54%) of trained SHP were retained in the same facility. Average trained staff transfer rate was 43%, with Uasin Gishu lowest at 24% and Garissa highest at 50%. Considering a subset of trained staff from level 4/5 facilities with distinct hospital departments, only a third (36%) of them are still working in relevant maternity/newborn/gynaecology departments. There was a statistically significant difference in transfer rate by gender in Garissa, Vihiga and the combined 5 counties (*p* < 0.05). Interval from training in years (1 year, AOR = 4.2 (2.1–8.4); cadre (nurse/midwives, AOR = 2.5 (1.4–4.5); and county (Uasin Gishu AOR = 9.5 (4.6- 19.5), Kilifi AOR = 4.0 (2.1–7.7) and Taita Taveta AOR = 1.9 (1.1–3.5), p < 0.05, were significant determinants of staff retention in the maternity departments.

**Conclusion:**

Retention of EmONC trained SHP in the relevant maternity departments was low at 36 percent. SHP were more likely to be retained by 1-year after training compared to the subsequent years and this varied from county to county. County policies and guidelines on SHP deployment, transfers and retention should be strengthened to optimise the benefits of EmONC training.

## Introduction

Globally, estimated 295,000 maternal deaths occurred in 2017, 2.5 million newborns died in 2018, and 2.6 million stillbirths occurred in 2015 with 99 percent, 77 percent and 98 percent of them occurring in low- and middle-income countries (LMICs) respectively [1–3]. The majority of these deaths occurred around the time of childbirth [4]. Kenya continues to experience a high burden of maternal and newborn deaths above the global sustainable development goal (SDG) targets of less than 70/100,000 live births for maternal mortality and 12/1000 live births for newborn mortality respectively [5]. In Kenya, the maternal mortality ratio was estimated at 342/100,000 live births in 2017 [6], perinatal mortality (stillbirths and early neonatal deaths) rate at 29/1000 in 2014 and neonatal mortality rate reduced to 22/1000 live births in 2014 [7]. The first Kenya Confidential Enquiry into Maternal Deaths report (2017) showed that substandard care was identified in 9 out of 10 maternal deaths with three quarters of the deaths associated with health workforce related deficiencies in knowledge and skills in emergency obstetric care (EmOC). The most frequently identified health workforce related factors were delay in starting treatment (32.9%), inadequate clinical skills (28.1%) and inadequate monitoring (26.9%) [8].

The Lancet Every Newborn Series report shows that three million lives can be saved by 2025 if achievable interventions are scaled up to nearly universal coverage, and improving care at the time of birth gives a triple return on investment saving mothers, newborns and stillbirths [9]. Increased coverage and quality of preconception, antenatal, intrapartum, and postnatal interventions by 2025 could avert 71% of neonatal deaths (1·9 million), 33% of stillbirths (0·82 million), and 54% of maternal deaths (0·16 million) per year. Evidence has shown that the day of birth is the most dangerous for mothers and their babies, resulting in nearly half of maternal and newborn deaths and stillbirths and therefore priority and urgent attention should be paid to this birth day. Preterm birth, intrapartum complications, and infections are the leading causes of neonatal death. Interventions delivered around the time of birth – skilled care during labor and childbirth, emergency obstetrics and newborn care for obstetric complications plus immediate newborn care – have the greatest potential to avert 41% of neonatal deaths [10–12]. However, barriers to scale-up of skilled care at birth with the highest effect on mortality outcome exist especially in LMICs including finance (only four percent of donor funding allocated to newborn health) and workforce, especially midwives and nurses – insufficient numbers, inefficient skill mix, inequitable distribution, varying levels and quality of education and training programmes [13, 14]. The Every Newborn Series recommends urgent attention to reach every woman and every newborn baby, close gaps in coverage, and improve equity and quality for antenatal, intrapartum, and postnatal care, especially in the poorest countries and for underserved populations for a triple return on investment around the time of birth: averting maternal and newborn deaths and preventing stillbirths [13]. This is also emphasized in the State of the World Midwifery (SoWMy) report 2021 which indicates that for midwives to achieve their potential, urgent investments are needed in the midwifery workforce planning, management and regulation and the work environment. This includes their production and deployment [14].

The medical and surgical interventions necessary to prevent this loss of life are known, and most maternal and newborn deaths are in principle preventable. The SoWMy 2021 report indicates that fully educated, licensed and integrated midwives supported by interdisciplinary teams and an enabling environment can deliver about 90% of essential sexual, reproductive, maternal, newborn and adolescent health (SRMNAH) interventions across the life course, even though they account for less than 10% of the global SRMNAH workforce [14]. Improving access to midwifery (including family planning & interventions for maternal and newborn health), could avert 83 percent of all maternal deaths, stillbirths and neonatal deaths [15]. Evidence shows that availability and utilisation of EmONC reduces maternal and newborn mortality [12, 16]. Skilled health personnel (SHP), commonly referred to as skilled birth attendants, should be trained to have the required competencies [17], and should work as part of an integrated team of maternal and newborn professionals (including midwives, nurses, obstetricians paediatricians and anaesthetists) performing all the signal functions of emergency obstetric and newborn care (EmONC). Such care provided within an ‘enabling environment’ that includes drugs, supplies, appropriate policies and a functional referral system is likely to result in optimisation of the health and well-being of women and newborns [18, 19]. In addition to competent, well resourced, multi-disciplinary maternal and newborn health (MNH) teams, country-level workforce management is required to ensure optimal recruitment, distribution and retention of and supervision of health workers is essential to improve access to high-quality care [20].

Kenya is committed to reducing preventable maternal and newborn deaths through the inclusion of EmONC training in its national health sector strategic and investment development plans [21, 22] and Vision 2030 [23] in the attainment of universal health coverage (UHC) targets. It developed its first emergency obstetric care training curriculum in 2006 and this was rolled-out in 2009 in 10 level 5 hospitals in the country in 2009 under the Making it Happen (MiH) I program supported by Liverpool School of Tropical Medicine (LSTM). In 2012, a revised Ministry of Health Kenya emergency obstetric and newborn care curriculum was implemented by LSTM to cover 15 additional counties located in the former Nyanza, Western and Central provinces. In 2014, the MiH program expanded the coverage of the trainings across all the 47 counties of Kenya. The MiH program had the objective to build the capacity of at least 80 percent of a multi-professional team of skilled health personnel (nurse/midwives, clinical officers and medical doctors including specialist obstetricians and paediatricians) working in the maternity units of health facilities to improve the availability and utilisation of EmONC and quality of maternal and newborn health outcomes.

Human resources for health, also known as the health workforce, is one of the building blocks proposed by the World Health Organization (WHO) Health Systems Framework and is central to achieving UHC and enhancing the achievement of the SDGs [24]. The proportion of births attended by SHP increased from 42% in 2003 to 62% in 2014 [7]. Despite this increase and the in-service EmONC trainings, the quality of maternal and newborn care provision is still sub-optimal [8]. Evidence on the status of EmONC services in Kenya has largely concentrated on the readiness and availability of these in health facilities, specifically focusing on equipment and supplies [25–28]. The results of three annual health facility assessments on availability and quality of EmONC services in Kenya between 2014 and 2016 in 18 counties showed that availability of equipment to perform all seven basic EmOC (BEmOC) signal functions (Table 1) increased progressively over the three annual assessments. In 2016, essential items for performing all seven BEmOC signal functions were available in 28 percent of health centres and dispensaries (a 14-fold increase from 2014) and 54 percent of hospitals (a two-fold increase from 2014) [26]. Little is documented on the availability of the trained SHP to provide essential EmONC services in the health facilities and poor staff retention after training may be reducing the optimal effect of the SHP strategy under the Ending Preventable Maternal Mortality global strategy and the Kenya strategic health sector development plan. For progress towards achievement of SDG targets, high quality health systems should focus on the population’s health needs and expectations, governance of the health sector and partnerships across sectors, platforms for care delivery, workforce numbers and skills, and tools and resources, from medicines to data [29].

**Table 1:** Emergency obstetrics and newborn care (EmONC) signal functions

BEmOC signal functions
(1) Administer parenteral antibiotics
(2) Administer uterotonic drugs (i.e., parenteral oxytocin)
(3) Administer parenteral anticonvulsants for preeclampsia and eclampsia (i.e., magnesium sulphate)
(4) Manually remove the placenta
(5) Remove retained products of conception (e.g., manual vacuum aspirations, misoprostol for medical evacuation)
(6) Perform assisted vaginal delivery (e.g., vacuum extraction, forceps delivery)
(7) Perform basic neonatal resuscitation (e.g., with bag and mask)
**CEmOC signal functions**
Perform all seven components of BEmOC, plus the following:
(8) Caesarean section
(9) Blood transfusion

*BEmOC* Basic emergency obstetrics care, *CEmOC* Comprehensive emergency obstetrics care

The objectives of this study were to determine the availability and staff transfer rates of SHP trained in EmOC, and to identify the determinants of their retention within maternity units up to five years after the training to inform local and national solutions to optimise the impact of EmONC trainings.

## Methods

### Study Design

This was a cross-sectional review of EmONC trained SHP in five counties (Kilifi, Taita Taveta, Garissa, Vihiga and Uasin Gishu) supported by LSTM for a 5-year period from 2014 to 2019. Data collection was conducted between January–February 2020. Secondary data was extracted from an LSTM maintained in-service EmONC training database. The original data was not collected for research purposes but part of routine EmONC training administrative and registration purposes. No primary data was collected.

### Study Setting

**Fig. 1:**
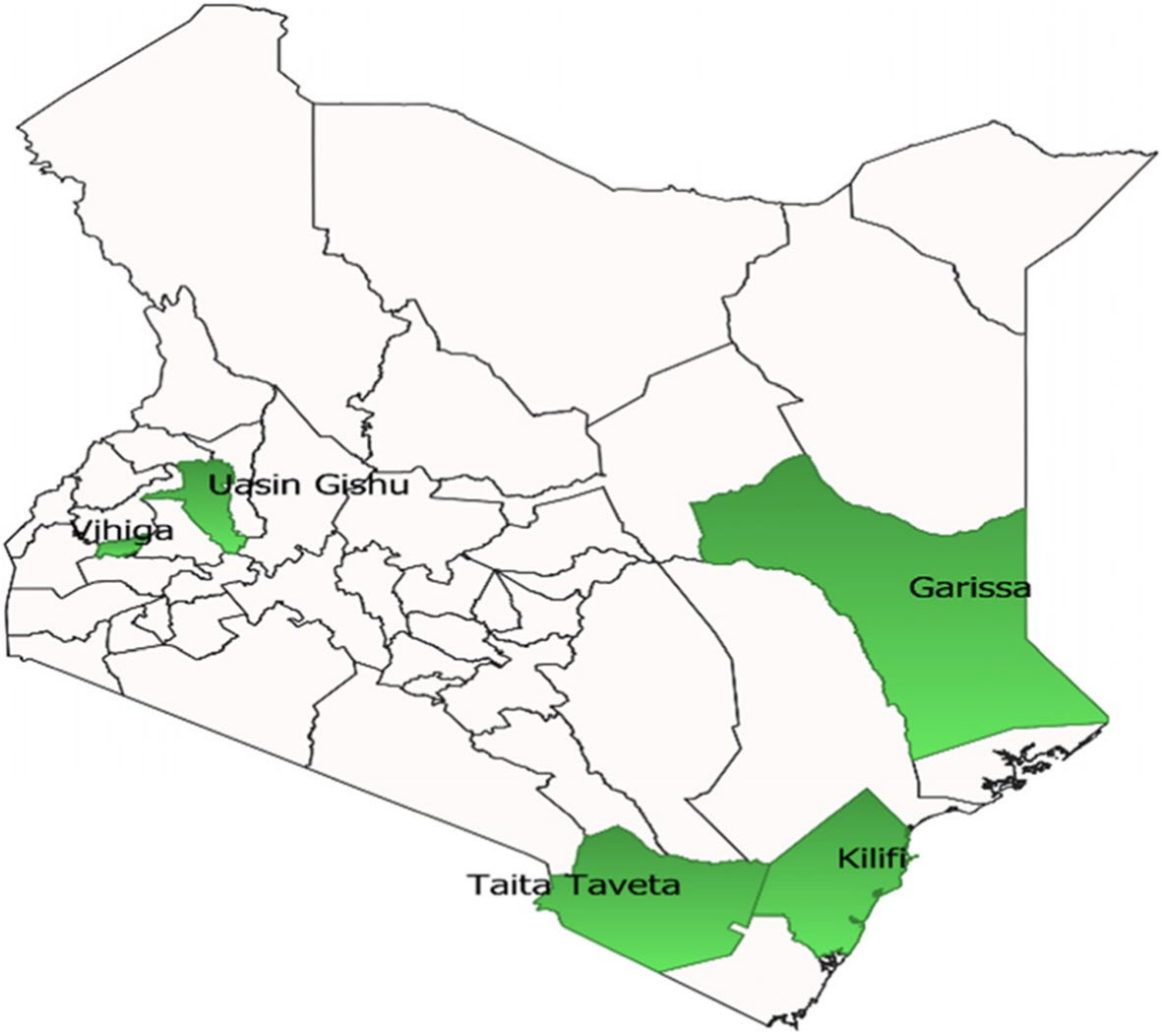
Five counties supported by LSTM included in the study

The assessment was conducted in the five counties (Kilifi and Taita Taveta from the coast, Garissa from the North Eastern Kenya, Vihiga from Western Kenya and Uasin Gishu from Rift Valley) supported by LSTM (Fig. 1). These counties in collaboration with LSTM conduct in-service EmONC trainings and other capacity building initiatives using a sustainable cost-sharing model. In this model, counties budget for and implement EmONC capacity building activities in their annual workplans. Multidisciplinary hands-on training within existing training facilities were organised without compromising the provision of services. Training and capacity building initiatives in EmONC target SHP working in maternity departments (mother and child health (MCH) clinics, maternity units, newborn units & gynecology units. Garissa and Kilifi were two of the 15 counties in Kenya with the highest burden of maternal mortality in 2014 [30]. Each of the counties has a single county referral hospital (except Uasin Gishu that has the national Moi Teaching and Referral Hospital) in addition to subcounty hospitals/faith-based/private hospitals, health centres and dispensaries providing basic and comprehensive emergency obstetrics and newborn care as appropriate according to the level, infrastructure and staffing levels of the facilities as per the minimum package of MNH services at different levels [31].

## Data collection

Data was extracted from an LSTM maintained in-service training database (staff gender, cadre, year trained, hospital level, and hospital location-sub-county or county). Verification of current health facility in which trained SHP were deployed was confirmed using a specifically designed data collection tool by the respective county reproductive health departments (in collaboration with the subcounty reproductive health coordinators and the facility in-charges) and the study team. The data collection tool captured information about whether the SHP was still working at the same facility they were working in when they were trained in EmONC during the study period and still working in maternity department. If they were not still working in the training facility, the reasons for this (transferred, resigned, went for further education, retired or died) were documented.

## Variables and measurements

The dependent variable was retention in maternity department, which was a binary outcome (yes or no). Retention in maternity department referred to a staff working in any of the following sections: mother and child health (MCH) clinics, maternity units (antenatal, intrapartum and postpartum), newborn units & gynecology units where they provide at least one EmONC signal function. The independent variables were: gender (male, female), cadre (nurse/midwife, medical doctor or clinical officer), interval from training in years (1, 2, 3, 4, 5 years), type of facility (dispensary, health centre, subcounty/county hospital) and county (Kilifi, Uasin Gishu, Taita Taveta, Garissa and Vihiga). Other variables (binary response; yes/no) included – staff still working at training facility, still working in maternity department, transferred, resigned, retired, died or unknown.

### Data analysis

Data was entered in Microsoft Excel 2016, cleaned and responses coded as binary (yes = 1 and no = 0). This was then exported to StataCorp. 2011. Stata Statistical Software: Release 12. College Station, TX: StataCorp LP. for statistical analysis. Descriptive data analysis – frequencies and proportions of EmONC trained SHP and transfer rate by county were calculated. The association between staff transfers and gender in the counties was tested through two-sample test of proportions and p-values less than 0.05 were considered statistically significant. Associations between independent variables (gender, cadre, hospital level, county and interval from training in years) and the dependent/outcome variable (retention in maternity department) were tested using binary logistic regression to identify the determinants of EmONC trained SHP retention. Odds ratios, 95% confidence intervals and p-values were reported. Independent variables with p-values less than 0.05 were considered statistically significant as determinants of EmONC trained SHP retention.

## Results

### Distribution of EmONC trained healthcare workers

A total of 927 SHP were trained in the five counties between 2014 and 2019. Most trained SHP were nurse/midwives (677, 73%) followed by clinical officers (151, 16%) and medical doctors (91, 11%). At the level of facility, the majority of the SHP (621, 67%) were from the hospitals, followed by health centres (219, 24%) and dispensaries (87, 9%) (Table 2).

**Table 2:** Distribution of EmONC trained SHP per level of facility per county

Facility level	Garissa	Kilifi	Taita Taveta	Uasin Gishu	Vihiga	Total	Total %
Dispensary (level 2)	47	35	5	0	0	87	9
Health centre (level 3)	66	56	38	4	55	219	24
Hospital (level 4/5)	241	91	86	101	102	621	67
Total	354	182	129	105	157	927	100%

Overall, 427 (46.0%) were still working in the facility where they worked when they received the EmONC training, 394 (42.5%) had been transferred to other health facilities, 63 (6.8%) had resigned and the reason for non-retention was not known for 32 (3.5%) EmONC trained SHP. Uasin Gishu county had the highest proportion of trained SHP still working in the same facility as when they were trained in EmONC, (72.4%) with Vihiga county having the lowest at 28.7%. Regarding staff transferred, in Garissa county half (50.3%) of its trained SHP had been transferred to other facilities while Vihiga had the highest proportion of trained SHP resigning (28%). Kilifi and Taita Taveta counties had 7.7% and 13.2% of the trained SHP with unknown status as their availability could not be accounted for by the health facilities (Table 3). A total of 26 SHP were in school for further education with the majority (16, 62%) being doctors followed by nurse/midwives (8, 31%) and clinical officers (2, 8%). Overall, 18% (16 of 91) of the doctors, one percent each for nurse/midwives (8 of 677) and clinical officers (2 of 151) trained were pursuing further education at the time of the assessment.

**Table 3:** 5-year status of EmONC trained SHP in the five counties

County	Garissa	%	Kilifi	%	Taita Taveta	%	Uasin Gishu	%	Vihiga	%	Total	%
Still in training facility	158	44.6	96	52.7	52	40.3	76	72.4	45	28.7	427	46.0
Transferred	178	50.3	66	36.3	59	45.7	25	23.8	66	42.1	394	42.5
Resigned	14	3.9	1	0.6	0	0	4	3.8	44	28	63	6.8
Retired	1	0.3	5	2.7	1	0.8	0	0	1	0.6	8	0.9
Died	2	0.6	0	0	0	0	0	0	1	0.6	3	0.3
Unknown	1	0.3	14	7.7	17	13.2	0	0	0	0	32	3.5
**Total**	**354**	**100**	**182**	**100**	**129**	**100**	**105**	**100**	**157**	**100**	**927**	**100**

### Period of training

Overall, the distribution of SHP per year of EmONC training was highest in 2019 (207) and lowest in 2015 (161) with no major differences between the years for the combined counties. However, variations existed across the counties with Garissa receiving at least one training course in each of the five years while Kilifi, Uasin Gishu and Vihiga received three training courses each in the 5-year period. No EmONC training course was supported in 2018 across any of the five counties (Table 4).

**Table 4:** Period of EmONC training and number of SHP trained in the 5 counties between 2014 – 2019

**County/year**	2014	2015	2016	2017	2019
Garissa	128	63	67	64	32
Kilifi	-	-	38	80	64
Taita Taveta	47	23	33	26	
Uasin Gishu	-	-	59	6	40
Vihiga	11	75	-	-	71
**All 5 counties**	186	161	197	176	207

### Association between staff transfers and gender per county

There was a statistically significant difference in staff transfer rate by gender in Garissa and Vihiga counties with more male SHP (58%) transferred in Garissa compared to females (42%), P = 0.0015 with the reverse for Vihiga with more females (47%) compared to males (27%), P = 0.0109. There were no statistically significant differences in the transfers by gender for Kilifi, Uasin Gishu and Taita Taveta counties (P > 0.05) (Table 5).

**Table 5:** Association between staff transfers and gender per county

COUNTY	MALE			FEMALE			*P*-VALUE
	Total trained	Transferred	% Transferred	Total trained	Transferred	% Transferred	
Garissa	189	109	58	165	69	42	0.0015*
Kilifi	53	19	36	129	47	36	0.4703
Taita Taveta	39	17	44	90	42	47	0.3737
Uasin Gishu	23	6	26	82	19	23	0.3858
Vihiga	41	11	27	116	55	47	0.0109*

^*^P < 0.05 statistically significant

### Retention of SHP in maternity departments

Data from hospitals was analysed as SHP in these are facilities are deployed to serve in distinct service departments (e.g. maternity department, medical/surgical department etc.), as opposed to health centres and dispensaries where there are no distinct departments and SHP work across all the departments in most cases, usually under one roof. Overall, only 223 (36%) of the trained SHP were still working in maternity departments. This varies significantly across counties with Uasin Gishu county having the highest staff retention rate of 73% while Garissa had the lowest retention rate of 19% (Table 6).

**Table 6:** 5-year staff retention in maternity departments per county

County	Working in Maternity department	Total Trained	% Working in Maternity department
Garissa	46	241	19
Kilifi	49	91	54
Taita Taveta	24	86	28
Uasin Gishu	74	101	73
Vihiga	30	102	29
**Total**	**223**	**621**	**36**

### Determinants of staff retention for EmONC trained SHP in maternity departments

County, cadre of staff and interval period from training were statistically significant determinants for staff retention in maternity departments. Compared to clinical officers and doctors, nurse/midwives were 3 times more likely to be retained in maternity departments (AOR 2.5, 95%CI 1.4—4.5, P < 0.0001). Compared to Garissa and Vihiga counties, EmONC trained SHP were 10 times in Uasin Gishu (AOR 9.5, 95%CI 4.6—19.5, P < 0.0001), four times in Kilifi (AOR 4.0, 95%CI 2.1—7.7, P < 0.0001) and twice in Taita Taveta (AOR 1.9, 95%CI 1.1—3.5, P = 0.032) more likely to be retained in maternity departments. Skilled health personnel were four times more likely to be retained in maternity departments during the first year after EmONC training (AOR 4.2, 95%CI 2.1—8.4, P < 0.0001). However, there was no association between 2 – 5 years interval since EmONC training and staff retention in maternity departments in the five counties (P > 0.05). Besides, there was no association between gender and staff retention in maternity departments (P > 0.05) (Table 7).

**Table 7:** Regression analysis for determinants of EmONC trained staff retention in maternity departments in the five counties

Factor	AOR	95% C.I		*P*-value
		Lower	Upper	
Gender				
Female (Ref)	1.0			
Male	0.8	0.5	1.2	0.213
Cadre				
Clinical Officer (Ref)	1.0			
Nurse/Midwife	2.5	1.4	4.5	< 0.0001*
Medical Doctor	0.9	0.4	1.9	0.784
County				
Garissa (Ref)	1.0			
Uasin Gishu	9.5	4.6	19.5	< 0.0001*
Kilifi	4.0	2.1	7.7	< 0.0001*
Vihiga	0.9	0.5	1.8	0.871
Taita Taveta	1.9	1.1	3.5	0.032*
Interval Period				
2014 (year 5) (Ref)	1.0			
2015 (year 4)	1.4	0.7	2.8	0.392
2016 (year 3)	1.0	0.5	2.1	0.973
2017 (year 2)	1.4	0.7	2.8	0.321
2019 (year 1)	4.2	2.1	8.4	< 0.0001*

^*^P < 0.05 statistically significant, *AOR* Adjusted odds ratio, *CI* Confidence interval

## Discussion

The purpose of this study was to identify the determinants of staff retention after EmONC training in Kenya. The findings will be useful to improve the availability and performance of all the EmONC signal functions and improve the quality of maternal and newborn health services and outcomes at health facilities.

Overall, our findings demonstrate that although practices on staff deployment and transfers varied across counties, overall retention of EmONC trained SHP in maternity departments was low (36%) in the five counties. Nurse/midwives had the highest odds of being retained in the maternity departments five years after the EmONC training compared to clinical officers and doctors. Besides, only SHP trained within the last year had a higher chance of retention in maternity departments compared with those trained 2 – 5 years ago.

For a return on investment, in-service trained SHP can demonstrate greatest impact in performance and health outcomes by utilising the acquired knowledge and skills when retained in related departments/wards/units where they can apply the skills. High levels of attrition—defined broadly as exits from the workforce, which may be due to emigration, voluntary exits (e.g. to other sectors of employment), illness, death or retirement – lead to a large loss of public resources spent on in-service training of a health worker [32]. To plan effectively for the future, more focus needs to be dedicated to the issue of workforce retention and attrition following in-service training.

Midwives educated and regulated to international standards and working in well-equipped enabling environments are able to provide the full scope of interventions needed when they are fully integrated into a well-functioning health system within a multidisciplinary team to ensure availability of quality MNH and referral services when required for pregnancy and childbirth complications and emergencies [33, 34]. Following the skilled health personnel definition by WHO and others, it is critical for trained competent integrated teams working within an enabling environment to be available in maternity departments for pregnancy and childbirth services [35]. Our study shows that midwives are better retained compared to other cadres. While retention of nurse/midwives can be improved, retention of all the team is essential. Redeployments and staff movements or rotations may be inevitable due to shortages and inequitable distribution in these low resourced settings and so a system to ensure that those available are trained to maintain competency is required.

The overall average staff transfer rate of 43% compares with the overall annual attrition rate of between 3 and 44% from a rapid review of attrition rates of health workers from the workforce in different regions and settings by Lopes and colleagues [36]. This high transfer rate is not beneficial as the investments made for in-service training a SHP in key skills relevant to improving practice and outcomes in a distinct department is likely not to result in the desired impact. As a result, there is need for harmonised local practices and interventions including local staff deployment policies and guidelines at county and facility level to ensure that trained SHP with key skills are retained in departments they can utilise their competencies for improved health outcomes.

Our study showed that a significant proportion of doctors were pursuing further education compared to the other cadres. This could pose short to medium term challenges to the health facilities but represents a good long term investment in pursuing specialist training especially in these low resourced and inequitably distributed human resources for health settings [37, 38]. Evidence has also shown that attrition contributes to increased workload and worse working conditions for the remaining workforce, which in turn contributes to lower quality of care and worse health outcomes [39]. This calls for a robust capacity building approach to strengthen the skills of the remaining SHP serving in the health facilities at any given time to allow for a seamless transition when personnel leave for specialist training. This may include a mandatory and periodic EmOC training, including as part of induction of new members of the maternity care team, mentoring of all staff in order to maintain good quality of care.

Our study had strengths and limitations. To the best of our knowledge, this is the first study in Kenya to identify the determinants of in-service staff retention in maternity departments after EmONC training. Our study catalysed the funding and implementation of a project to improve the capacity of sub national health managers to monitor and improve staff retention after EmONC training. Specifically, the goal was to improve the capacity of County Health Management Teams (CHMT)/ government health facilities’ managers to manage retention of trained SHP in relevant departments for enhanced return on training investment and for improved maternal and newborn health outcomes. The project interventions were (1) a review of human resource management practices including maintaining a skills training database and development of healthcare workers’ dashboard at the health facilities, sub-counties and counties included in this study for future planning for deployment, capacity building and transfers/rotations based on specific skillset for greatest impact on quality of care and (2) the drafting and implementation of a declaration of intent – a signed agreement between health facilities and county health managers whose goal was to improve MNH health outcomes by committing to intentional transfer of EmONC trained staff and a sustainable training approach supported by facility and county health management. The intended outcome of the project was that policy makers and health facility managers have capacity to ensure that all maternity care staff are trained in EmONC at any given time, supported by at least one trained mentor per facility. Our study did not examine annual trends in retention/transfers and whether the transfers are to relevant departments where healthcare workers can demonstrate the greatest impact either intra-facility, intra-county or inter-facility and intercounty. As time goes by after training, more staff moving either within or between health facilities or departments is not surprising – whether due to personal, higher education, logistical reasons or career advancement/promotion. However, our study did not establish who decides the movement of the trained staff – whether by staff choice or by authorities. Use of secondary data – the training registration data captured misses out on other important determinants for staff turnover and transfers such as healthcare worker’s personal (physical, mental – emotional and social including marital status and other family responsibilities), job satisfaction at the current position, social support – opportunities for career development, stress at the current position and organisational factors (reward, salary and other benefits) which would have provided a deeper understanding of the key determinants for staff turnover in the maternity departments and health facilities [40–42]. Future research focusing on primary data collected from healthcare workers, health facility and county health managers should integrate this data to establish salient factors contributing to poor staff retention following investments in building the capacity of in-service health workforce.

Our findings have implications for practice and policy. For a return on investments and impact, counties and health facilities should strengthen capacity building and retention strategies of all skilled health personnel with EmONC skills in maternal and newborn health. Health managers should develop and implement local strategies, policies and protocols for staff deployment/transfers/replacement and retention based on key competencies by SHP and maintain a skills training database as well as provide an enabling working environment and motivation for SHP to apply the acquired competencies to prevent unnecessary attrition as well as preventing occupational stress [43–46]. Further research is needed to examine the annual trends in SHP transfers and retention in relevant departments. In addition, a system should be in place to monitor and investigate the SHP transfer or retention, maintain a staff training database, utilize and implement a staff training dashboard and the association between trained staff retention and MNH outcomes should be monitored regularly by facility managers.

## Conclusion

Retention of EmONC trained SHP in the relevant maternity departments where they can demonstrate the greatest impact was low at 36 percent. Two out of five (42.5%) EmONC trained SHP were transferred to other health facilities post-training. SHP were more likely to be retained during the first year after training compared to the subsequent years and varied from county to county. County policies and guidelines on SHP deployment and transfers/retention should be strengthened to optimise the benefits of EmONC training. The study improved the capacity of sub national health managers to monitor and improve staff retention after EmONC training through a skills training database, development of healthcare workers’ dashboard to monitor training needs and deployments/transfers and declaration of intents between health facilities and counties advocating for and committing to a sustainable staff training and retention in areas where they can demonstrate greatest impact. Future studies to examine the effect of staff transfers or retention on the maternal and newborn health practices and outcomes in the health facilities as well as whether the staff movement is by choice or by the authorities, and other individual or institutional factors through participant interviews are needed.

## Data Availability

The datasets used and/or analysed during the study are available from the corresponding author on reasonable request.

## Abbreviations

ANC: Antenatal care
AOR: Adjusted odds ratio
BEmONC: Basic emergency obstetrics and newborn care
CEmONC: Comprehensive emergency obstetrics and newborn care
CI: Confidence Interval
EmONC: Emergency Obstetrics and Newborn Care
EOC: Emergency obstetrics care
LSTM: Liverpool School of Tropical Medicine
MiH: Making it Happen
MNH: Maternal and newborn health
MOH: Ministry of Health
SDG: Sustainable development goal
SHP: Skilled health personnel
SoWMy: State of the World Midwifery
WHO: World Health Organization
